# Identification and Characterisation of Endophytic Bacteria from Coconut (*Cocos nucifera*) Tissue Culture

**DOI:** 10.21315/tlsr2020.31.1.4

**Published:** 2020-04-07

**Authors:** Elv Nhiel Salo, Annabelle Novero

**Affiliations:** Department of Biological Sciences and Environmental Studies, College of Science and Mathematics, University of the Philippines Mindanao, Tugbok District, Davao City 8022, Philippines

**Keywords:** Endophyte, Coconut, 16s rDNA analysis, Phylogenetics, *Pantoea dispersa*, *Bacillus subtilis*

## Abstract

The coconut is an important economic crop in the Philippines which currently ranks as the world’s second largest producer. This study characterised and identified endophytes from coconut tissue culture in order to gain an initial understanding of their potential uses as sources of bioproducts. The isolates were evaluated using morphological, biochemical and molecular methods. Gram staining results revealed that four out of five bacteria isolated were Gram positive. Isolate CEB 1 fermented all three sugars in the Triple Sugar Iron Test while the other four did not. 16S rDNA gene fragments were amplified from genomic DNA using the universal primers 16F27 and 16R1542. The 16S rDNA sequence were found to be homologous to *Bacillus subtilis* and *Pantoea dispersa.* Phylogenetic analyses showed significant clustering of bacterial isolates together with archived DNA of *B. subtilis* and *P. dispersa.* All isolated bacteria matched the characteristics of their molecular homologies. Isolate CEB 5, identified as *B. subtilis*, produced red pigments which are possibly pulcherrimin. Literature reports that pulcherrimin possesses antimicrobial activity against yeast species, microscopic fungi, and postharvest pathogens. *P. dispera*, on the other hand, has been reported to convert insoluble phosphorus into soluble form to enable plants to take up more phosphorus. Determination of the bioactivities of endophytes reported in this study may enable the discovery of novel bioproducts.

**Highlights**

- Five bacterial species were isolated and identified from coconut tissues.
- Morphological, biochemical and molecular analyses revealed that four of the isolates were different strains of *Bacillus subtillis*.
- The fifth isolate was identified as *Pantoea dispersa*.

## INTRODUCTION

The Philippines is the second largest producer of coconut accounting for 32.67% of world production (Naik 2017). The traditional way of planting coconut is by seed. Coconut palms planted from seeds take about five to six years before the first fruits develop (Chan & Elevitch 2006). This makes the replacement of palms very time consuming in terms of coconut production since it would take time before plants could produce fruit. To remedy this, micropropagation of coconut has been adapted through coconut somatic embryogenesis technology (Luis *et al*. 2012; Ree & Guerra 2015).

Many problems are encountered during the course of micropropagation. Examples of these would be phenolic exudation, necrosis, habituation and contamination (Bhatia *et al*. 2015). Contamination is defined as “accidental introduction of undesirable bacterial, fungal or algal microorganisms” (Bhatia *et al.* 2015). Contamination may be chemical or biological. Chemical contaminants come from the reagents and materials used while biological contaminants come from the plant itself. The presence of contaminants may result to loss of time, money, effort and valuable products (Ryan *et al*. 2008). However, contaminants present may in fact be endophytes.

Endophytes are microorganisms that reside inside the plant tissues. They are traditionally assumed to be latent pathogens that do not trigger harmful reactions or disease symptoms and provide no benefit to the host plant (Zinniel *et al.* 2002). Endophytes are symbiotic microorganisms that infect the interior plant tissues without causing any pathogenic infections (Schulz & Boyle 2006). A large number of experimental evidences demonstrated that bacterial endophytes support the plant growth, development and yield by synthesizing different plant hormones (Figueiredo *et al.* 2009). Endophytes are protected from environmental stresses and microbial competition by the host plant tissue and seem to be ubiquitous in plant tissues (Kobayashi & Palumbo 2000).

In plant tissue culture, contaminants may persist despite the surface sterilisation of tissues. This causes a huge risk to the health of the explants since they compete with the plant in nutrients on the medium (Labrador *et al*. 2014). However, these endophytes may be endophytic fungi or bacteria which have specific roles in the growth of the plant. Labrador *et al.* (2014) reported a few endophytic bacteria isolated and characterised from sago palm (*Metroxylon sagu* Rottb.) tissue culture. These endophytic bacteria isolated from sago palm may probably be also present in coconut since they are both members of Family Arecaceae. Three bacterial species identified by Labrador *et al.* (2014) were members of Phylum Proteobacteria, Class Gammaproteobacteria, Family Enterobacteriaceae. The bacteria possess similar physiochemical characteristics in that they were all Gram-negative bacilli and performed similarly in biochemical tests conducted. They were all able to produce catalase and utilise citrate as a carbon source but were not able to produce tryptophanase. They were able to use the 2, 3-butanediol pathway but not the mixed acid pathway. They were also able to utilise glucose, lactose and sucrose and produce gas as well.

This study isolated and characterised endophytic bacteria in coconut tissue culture so that their possible beneficial role to the plant may be elucidated. Potential bioactive compounds present in the endophytes may also be identified and put to other uses in the future.

## MATERIALS AND METHODS

### Isolation of Endophytic Bacteria

This study was conducted from to April 2017 to May 2018. Five bacterial samples from coconut plumule explants in tissue culture were collected and plated on nutrient agar (NA) medium composed of 3 g beef extract, 5 g peptone, 5 g NaCl_2_ and 8 g agar per litre of water. The bacteria were purified via repetitive streaking on NA plates. Pure cultures of bacteria for biochemical tests were stored on fresh NA slants. Bacterial isolates were duly designated as CEB (coconut endophytic bacteria) 1 to 5.

### Morphological and Biochemical Characterisation

Macroscopic features of the isolated bacterial colonies were assessed with the following criteria: Colour of colony, elevation, margin, opacity of the colony, consistency, and surface of the colony. Microscopic features were determined through Gram staining. For the Gram test, a bacterial smear was prepared by mixing an isolated bacterial colony with a drop of distilled water on a glass slide. This was then air-dried and quickly passed on an open flame three times in order to heat-fix the bacterial smear. A small amount of ammonium oxalate crystal violet just enough to completely cover the smear was added and allowed to settle for 60 s. After 60 s, the stain was gently washed off with distilled water and a sufficient amount of Gram’s iodine, as mordant, was added. After 60 s, the mordant was again gently washed off with water and was decolourised by flooding the slide with 95% ethanol for 15 s. After which, the EtOH was removed with distilled water and a sufficient amount of safranin was added and allowed to stain for 30 s. The stain was then washed away with distilled water before microscopic examination.

Endospore formation was assessed by heat-fixing bacterial smear preparations. Each slide containing the bacterial smear was covered with layers of tissue paper and was placed on the staining rack, which was positioned on top of a pan with boiling water. The covered slide was flooded with malachite green and steamed for 7 min. Afterwards, the paper cover was removed and the slide was rinsed and stained with 0.5% safranin. The appearance of green spores and red vegetative cells indicate a positive result in microscopic examination.

Biochemical tests (catalase, citrate and triple sugar iron test) were performed. For the catalase test, a drop of 3% hydrogen peroxide (H_2_O_2_) was added to the glass slide. A colony of bacterial isolate from NA plates was then mixed on the glass slide. An immediate evolution of bubbles indicated a positive result (Cappuccino & Sherman 2014). *Staphylococcus aureus* was used as a positive control.

For the citrate test, isolates were inoculated onto Simmons citrate agar slants then incubated for 24 h at 37°C. A change in media color from green to blue, along with the presence of growth, indicated a positive result (Cappuccino & Sherman 2014; Labrador *et al*. 2014). *Pseudomonas aeruginosa* was used as a positive control.

Isolates were streaked onto Triple Sugar Iron agar slants. The three sugars were lactose, sucrose, and glucose. The inoculated slants were then incubated for 24 h at 37°C. After incubation, the agar slants were observed for changes in colour of the media on the butt and slant (Cappuccino & Sherman 2014), presence or absence of gas formation, and H_2_S production. *Salmonella typhi* was used as a positive control.

### Molecular Characterisation

Bacterial DNA was extracted using Trizol Reagent (Invitrogen, Thermo Fischer Scientific, USA) following the manufacturer’s protocol. In a sterile microcentrifuge tube, approximately 0.25 mL (1 × 10^7^ cells) of the bacterial pellet was placed. After quickly mixing in a vortex, the sample was centrifuged (5 min at 958 × g at 4°C). The supernatant was removed and 1 mL of TRIzol™ Reagent was added to the bacterial pellet. The solution was homogenised by gently pipetting it up and down. The sample was added with 0.2 mL of chloroform, gently mixed by inversion and incubated for 3 min. The sample was then centrifuged (15 min at 12,000 × g at 4°C). After centrifugation, the interphase containing the DNA was then separated from the lower phenol-chloroform and the upper aqueous phase and then placed in a clean, sterile microcentrifuge tube. 300 μL of absolute ethanol was added in the microcentrifuge tube and mixed by gently inverting the tube several times. The sample was then incubated for 3 min and centrifuged (5 min at 2000 × g at 4°C). After removing the supernatant, the sample was washed using 1 mL of 0.1 M sodium citrate in 10% ethanol (pH 8.5) with an incubation time of 30 min. After incubation the sample was centrifuged (5 min at 2000 × g at 4°C). The supernatant was removed. After this, 1.5 mL of 75% ethanol was added to resuspend pallet After 20 min incubation, the sample was centrifuged (5 min at 2000 × g at 4°C) and the supernatant was removed. The DNA pellet was allowed to dry in a laminar flow hood and then resuspended in 50 μL of TE buffer.

The bacterial 16S rDNA, which is about 1.5 kb long, was amplified using the primers: Forward, 16F27 (5’-AGAGTTTGATCCTGGCTCAG-3’) and Reverse, 16R1542 (5’-AAGGAGGTGATCCAGCCGCA-3’) (Gurtler & Stanisich 1996). A 2x *Taq* master mix (Vivantis, USA) containing: *Taq* DNA Polymerase (0.05 U/μL), 2x Vibuffer A, 0.4 mM dNTPs and 3.0 mM MgCl_2_ was used. Polymerase chain reaction (PCR) amplifications were performed in a thermal cycler (Veriti Dx 96-well Thermal Cycler, Applied Biosystems, USA). The PCR conditions were adapted from Labrador *et al*. (2014): Initial denaturation (95°C, 2 min); 30 cycles of denaturation (95°C, 1 min); annealing (65°C, 1 min); and extension (72°C, 1.5 min); and lastly, final extension (72°C, 5 min). The PCR products were run in agarose gel electrophoresis. Amplicons were excised and purified using a DNA the GF-1 Ambiclean DNA Recovery Kit (Vivantis, USA). Amplicons were sent to Macrogen, South Korea for standard DNA sequencing. The chromatograms received were edited using FinchTV (Geospiza Inc., Seattle, USA). A contig sequence was constructed using Bioedit (Ibis Biosciences, California, USA). The sequences were then compared to a library of 16S rDNA sequences of various bacteria using Basic Local Alignment Search Tool (BLAST) analysis (Altschul *et al*. 1990).

The contig sequences were aligned using ClustalW (Thompson *et al*. 1994) in the MEGA6 (Tamura *et al*. 2011) software. After alignment, a phylogenetic tree showing the relationship between the bacterial isolates was constructed using the Neighbour-Joining Method (Tamura *et al*. 2011) using 1000 bootstrap replicates as suggested by Jasim et al. (2014) and Kumar *et al*. (2015) and Maximum Likelihood Method as suggested by Verstraete *et al*. (2013) using 100 bootstrap replicates.

## RESULTS AND DISCUSSION

Table 1 shows the results of the Gram staining, catalase test, citrate test, triple sugar iron slant, and endospore staining. Isolate CEB1 was observed as a Gram-negative coccobacillus. Coccobacillus bacterium has a shape which is an intermediate between coccus and bacillus bacteria. The shape may range from round to short rod (Madigan *et al*. 2012). CEB1 was shown to be catalase-positive but non-hydrogen sulphide producer. It was able to ferment all three sugars (lactose, sucrose and glucose). Biochemical test results implied that isolate CEB1 is a member of the Family Enterobacteriaceae. Results were not sufficient to identify it to the genus level.

Isolates CEB2, CEB3, CEB4, and CEB5 were Gram-positive bacilli arranged in chains. These four isolates were catalase-positive and were able to produce endospores. Furthermore, they were not able to grow in Simmons’ citrate agar. They were also not able to produce hydrogen sulphide and were not able to ferment glucose, sucrose and lactose.

**Figure 1:**
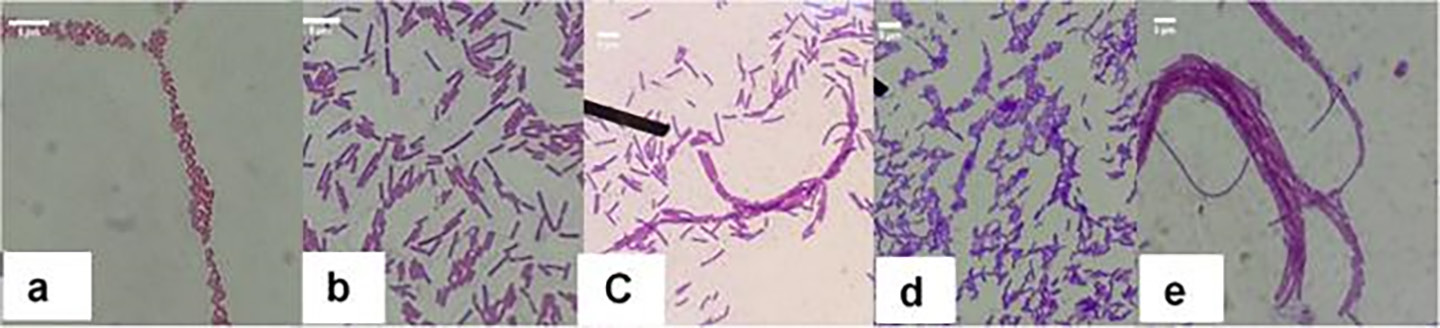
Photo micrographs of bacteria isolated from coconut at 1000X magnification; (a) CEB 1; (b) CEB 2; (c) CEB 3; (d) CEB 4; (e) CEB 5.

Based on the morphological characteristics and biochemical tests done, CEB 2, CEB 3, CEB 4, and CEB 5 are most likely species of the genus *Bacillus*. However, CEB 5 was observed to possess red pigmentation. Fig. 1 shows representative bacteria of CEB isolates. BLAST analysis of the deduced bacterial DNA sequences aligned isolate CEB 1 with the 16S rDNA sequence of *Pantoea dispersa* with 96 % identity while CEB 2, CEB 3, CEB 4 and CEB 5 aligned with the 16S rDNA sequences of *Bacillus subtilis* strains with 99% identity (Table 2). The assembled sequences ranged from 1255 to 1459 bp which fell within the expected nucleotide length of 16S rDNA fragments. The deduced sequences of CEB isolates were deposited in Genbank and were assigned corresponding accession numbers (Table 2).

**Figure 2:**
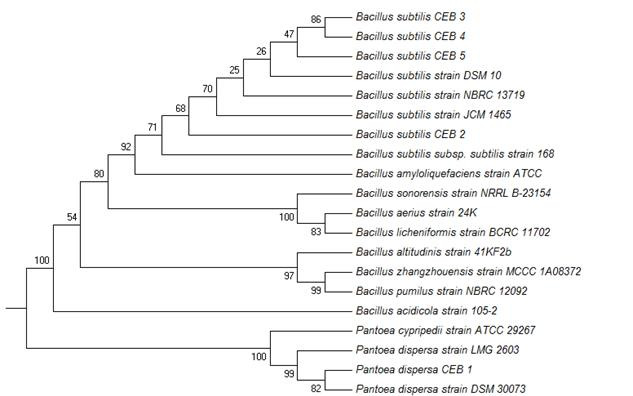
Phylogenetic relationships of CEB isolates with others already deposited in GenBank.

Phylogenetic analysis revealed that CEB 2, CEB 3, CEB 4, and CEB 5 were grouped together with *Bacillus subtilis* with a bootstrap value of 91% while CEB 1 was clustered with *P. dispersa* with a bootstrap value of 99% (see Fig. 2). CEB 2 branched off from the other bacillus isolates because CEB 2 is highly similar to *Bacillus subtilis* subsp. *subtilis* strain 168 compared to other strains of *Bacillus subtilis.*

The identity of CEB 1 as *P. dispersa* conformed with the study of Kalimutho *et al*. (2007) which reported that *P. dispersa* is a Gram-negative bacillus or coccobacillus having a yellow creamy colony appearance in synthetic sea water medium, fermenter of lactose and sucrose, do not produce H_2_S, and are positive for catalase and citrate utilisation. These descriptions are consistent with the characteristics of CEB 1 as discussed in this present study.

The genus *Pantoea* consists of many species which inhabit a wide range of environments (Walterson & Stavrinides 2015). It can be isolated in aquatic and terrestrial environments and can also be observed in a wide variety of host associations with plants, animals and insects. *P. dispersa* is commonly known as a pathogenic bacterium in plants such as sugar cane and grapes and in humans as well. However, there are many strains of *P*. *dispersa* which are epiphytic and endophytic (Walterson & Stavrinides 2015). Even though *P. dispersa* is part of a family of Gram-negative bacilli, various studies and literature have shown that *P. dispersa* may also occur as coccobacilli (Kalimutho *et al*. 2007; Krishnan *et al*. 2012; Berger 2018). Verma *et al*. (2017) reported that *P. dispersa* is a common endophyte found in rice seeds and inoculation of *P. dispersa* into antibiotic-treated seeds increased the root and shoot development of as well as recovery in root hair formation of seedling. This showed the importance of endophytic *P. dispersa* as a modulator of root and shoot development in rice seeds. Chen *et al*. (2014) reported that *P. dispersa* was isolated from cassava (*Manihot esculenta*) roots. Furthermore, this bacterium has been shown to facilitate the solubilisation of phosphate which enables host plants to acquire more phosphorus in red acidic soils. Phosphorus is an essential element required by plants for various biochemical processes. Therefore, they are required in large amounts. *P. dispersa* converts insoluble forms of phosphorus into soluble form so plants can acquire more phosphorus from the soil.

Molecular characterisation has identified the four isolates as *Bacillus subtilis.* The identity is consistent with the results of the catalase test and triple sugar iron test (Nakano *et al*. 1997). Even though *Bacillus subtilis* is commonly known to be citrate positive, a study by O’Donnell *et al*. (1980) has shown that there are strains of *Bacillus subtilis* that are citrate negative. Of all the four isolates, only CEB 5 was shown to produce a red pigment which makes it unique compared to the other *Bacillus subtilis* isolates, yet phylogenetic analysis has shown that it is grouped with CEB 3 and CEB 4 which are nonpigmented bacteria. The plausible reason for this is that the difference between CEB 5, CEB 3 and CEB 4 may not be found in the 16S rDNA gene but in other genes. CEB 5 may be a strain of *Bacillus subtilis* different from CEB 3 and CEB 4 but whole genome sequencing is necessary to verify if they are of different strains or not.

The red pigment produced by CEB 5 is believed to be pulcherrimin. The production of pulcherrimin was reported to be a result of the nonenzymatic reaction between iron in the nutrient agar and pulcherriminic acid excreted by the cells (Uffen & Canale-Parola 1971). According to Moeller *et al*. (2005), these pigments produced by *Bacillus subtilis* serve as protection against environmental radiation by shielding the sensitive spore components such as the DNA. Furthermore, pulcherrimin was reported to possess antimicrobial activity against yeast species, microscopic fungi, and postharvest pathogens as well (Kantor *et al*. 2015). In this study, the ability of CEB 5 to produce pulcherrimin was not verified. To verify if CEB 5 really produces pulcherrimin, identification tests (Cook & Slater 1954) can be done.

*Bacillus subtilis* is commonly found in the soil or on plant surfaces (Abd Allah *et al*. 2017). They are also considered as common endophytes found in various plants. In a study by Gond *et al*. (2015), *Bacillus subtilis* isolated from maize seeds were shown to produce lipopeptides which inhibit growth of a known fungal pathogen, *Fusarium monoliforme*. This study showed the antifungal properties exhibited by *B. subtilis* which prevent fungal infection of host plants.

Abd Allah *et al.* (2017) showed that *B. subtilis* alleviated the negative effects of high salt concentration in chickpea such as chlorophyll degradation, thereby improving the resistance of chickpeas in areas of high salt concentration.

From various literatures, *P. dispersa* and *B. subtilis* were known to provide beneficial effects to their host plants, from improving plant growth to protecting it against environmental stresses and pathogenic microbes. It would be interesting to examine how these endophytes interact with coconut in ways which are not only beneficial for coconuts themselves but for people who rely on coconuts as a source of income.

## CONCLUSION

The coconut is a crop important to the Philippine economy. Coconut production is beset by many problems such as the production of good quality planting materials. Tissue culture is a non-traditional approach by which coconut planting materials may be produced. In tissue culture, although microbial contaminants may hinder laboratory protocols, these microorganisms may also be beneficial endophytes. The successful identification of endophytes from coconut would allow the determination of bioproducts from these endophytes. Antimicrobial assays to test the activities of *B. subtilis* and *P. dispersa* identified in this study against plant pathogenic bacteria and fungi can be conducted. Tests for the identification of pulcherrimin production can also be done.

Aside from prospecting the beneficial effects of endophytes, elucidation of their identities and characteristics could aid tissue culturists on how to better manage these bacteria in tissue culture.

## Figures and Tables

**Table 1:** Biochemical characteristics of coconut endophytes.

Code	Gram classification	Shape	Arrangement	Catalese test	Triple sugar iron test	Citrate utilisation	Endospore formation
CEB 1	Gram negative	Coccobacilli	Single	+	A/A no H2S	+	n/a
CEB 2	Gram positive	Bacilli	Chains	+	K/K no H2S	−	+
CEB 3	Gram positive	Bacilli	Chains	+	K/K no H2S	−	+
CEB 4	Gram positive	Bacilli	Chains	+	K/K no H2S	−	+
CEB 5	Gram positive	Bacilli	Chains	+	K/K no H2S	−	+

**Table 2:** Molecular identities of coconut endophytes.

Isolate	Sequence length, bp	GenBank accession number	Homolog	Query cover (%)	Identity (%)	Reference
CEB 1	1459	MH_220244	*Pantoea dispersa* strain DSM 30073 16S ribosomal RNA gene, partial sequence (NR_116797.1)	100	96	Volksch *et al.* (2009)
CEB 2	1255	MH_220245	*Bacillus subtilis* subsp. *subtilis* strain 168 16S ribosomal RNA, complete sequence (NR_102783.2)	100	99	Barbe *et al.* (2009)
CEB 3	1439	MH_220246	*Bacillus subtilis* strain JCM 1465 16S ribosomal RNA gene, partial sequence (NR_113265.1)	100	99	Yarza *et al.* (2013)
CEB 4	1454	MH_220247	*Bacillus subtilis* strain DSM 10 16S ribosomal RNA gene, partial sequence (NR_027552.1)	99	99	Fritze & Pukall (2001)
CEB 5	1440	MH_220248	*Bacillus subtilis* strain NBRC 13719 16S ribosomal RNA gene, partial sequence (NR_112629.1)	100	99	Miyashita (2006)
